# Supplementary material for: Asynchronous magnetic resonance elastography: Shear wave speed reconstruction using noise correlation of incoherent waves
Source: Magn Reson Med. 2022 Oct 27;89(3):990–1001. doi: 10.1002/mrm.29502 (PMC9792433; doi:10.1002/mrm.29502)
Supplement: Supplementary file 1 — DATA S1 MATLAB code used to generate the numerical simulations of Figure 1 [file MRM-89-990-s002.zip › k-Wave/helpfiles/kspaceFirstOrder2DG.html]

kspaceFirstOrder2DG :: Functions (k-Wave)


# kspaceFirstOrder2DG

2D time-domain simulation of wave propagation on a GPU using C++ CUDA code.

## Syntax

See `kspaceFirstOrder2D` and `kspaceFirstOrder3DC`.

## Description

`kspaceFirstOrder2DG` provides a blind interface to the C++/CUDA version of `kspaceFirstOrder2D` (called `kspaceFirstOrder-CUDA`) in the same way as `kspaceFirstOrder3DC`. Note, the C++ code does not support all input options, and all display options are ignored (only command line outputs are given). See the k-Wave user manual for more information.

The function works by appending the optional input `'SaveToDisk'` to the user inputs and then calling `kspaceFirstOrder2D` to save the input files to disk. The contents of `sensor.record` (if set) are parsed as input flags, and the C++ code is run using the `system` command. The output files are then automatically loaded from disk and returned in the same fashion as `kspaceFirstOrder2D`. The input and output files are saved to the temporary directory native to the operating system, and are deleted after the function runs.

This function requires the C++ binary/executable of `kspaceFirstOrder-CUDA` to be downloaded from www.k-wave.org/download.php and placed in the "binaries" directory of the k-Wave toolbox (the 2D and 3D code use the same binary). Alternatively, the name and location of the binary can be specified using the optional input parameters `'BinaryName'` and `'BinariesPath'`.

## See Also

`kspaceFirstOrder2D`, `kspaceFirstOrder2DC`, `kspaceFirstOrder3DC`
